# Imbalance of Circulating B‐Cell Subsets Was Associated With Higher Risk of Relapses in RRMS Patients

**DOI:** 10.1155/jimr/7341319

**Published:** 2026-09-22

**Authors:** Lana Márcia Lopes, Marcos O. S. Cafasso, Joana Hygino, Priscila M. Sacramento, Marisa C. Sales, Taissa M. Kasahara, Larissa Cristine S. Lopes, Carolina Alvarez de Azevedo, Lendel C. da Costa, Átila D. Rossi, Bruno Francisco Teixeira Simões, Cleonice A. M. Bento, Claudia Cristina Vasconcelos

**Affiliations:** ^1^ Department of Microbiology and Parasitology, Federal University of the State of Rio de Janeiro, Rio de Janeiro, Brazil, ufrj.br; ^2^ Post-Graduate Program in Neurology, Federal University of the State of Rio de Janeiro, Rio de Janeiro, Brazil, ufrj.br; ^3^ Post-Graduate Program in Microbiology, University of the State of Rio de Janeiro, Rio de Janeiro, Brazil, uerj.br; ^4^ Post-Graduate Program in Computer Science, Federal University of the State of Rio de Janeiro, Rio de Janeiro, Brazil, ufrj.br; ^5^ Department of Genetics, Federal University of Rio de Janeiro, Rio de Janeiro, Brazil, ufrj.br

**Keywords:** B cells, CXCL13, IL-10, IL-17, MS, NfL

## Abstract

**Objectives:**

B‐cell aggregates below the meninges have been associated with progression of neurodegeneration in multiple sclerosis (MS). We aimed to investigate the frequency of different B‐cell subsets and their relationship with MS severity. Also, we quantified the CXCL13 and neurofilament (NfL) plasma levels.

**Methods:**

According to the differential expression of IgD, CD38, CD27, HLA‐DR, CD138, IL‐10, and IL‐17, the frequency of different circulating B‐cell (CD19^+^) subsets was evaluated using flow cytometry. Plasma levels of CXCL13 and NfL were quantified using ELISA.

**Results:**

Here, the severity of neurological impairment positively correlated with the percentage of plasmablast (CD138^+^, IL‐17^+^IL‐10^−^, HLA‐DR^+^IgD^−^) and memory CD27^+^CD38^+^B‐cell subsets (IL‐17^+^IL‐10^−^ and HLA‐DR^+^IgD^−^), but negatively correlated with the proportion of HLA‐DR^−^IgD^+^ and IL‐17^−^IL‐10^+^ among transitional and plasmablasts. The occurrence of new clinical relapses was mainly observed among patients who showed an elevated proportion of CD138^++^ and HLA‐DR^++^IgD^−^ plasmablast and HLA‐DR^+^IgD^−^ memory and CD27^+^CD38^+^B‐cell subsets. Further, relapsed patients showed a higher percentage of IL‐17^+^IL‐10^−^ cells among transitional, naïve and different memory B cells at baseline. By contrast, elevated frequency of HLA‐DR^−^IgD^++^ memory B cells and IL‐17^−^IL‐10^+^ among transitional and plasmablasts was observed in nonrelapsed patients over time. CXCL13 levels were higher in relapsed patients, and positively correlated with CD138^+^ plasmablasts and memory B‐cell subsets. Finally, plasma levels of NfL correlated positively with both CD138^+^ and HLA‐DR^+^IgD^−^ plasmablasts, but negatively with transitional and naïve B cells with HLA‐DR^−^IgD^+^ phenotype.

**Conclusions:**

In summary, our findings suggest that increased circulating anergic or immunogenic B‐cell subsets could be candidate biomarkers for low‐ and high‐risk of disease activity, respectively.

## 1. Introduction

In most patients diagnosed with multiple sclerosis (MS), the disease evolves via recurrent acute episodes of neurological impairment followed by total or partial clinical remission [1]. In this form, called relapsing–remitting MS (RRMS), relapses have been attributed to autoimmune attacks mainly coordinated by myelin‐specific Th17 cells [2–6]. Unfortunately, treatment of RRMS patients with disease‐modifying therapy (DMT), known to inhibit and/or block the access of T cells to the central nervous system (CNS), has little effect on the risk of disease progression to the secondary progressive form (SPMS). SPMS is characterized by progressive neurodegeneration with cortical atrophy even in the absence of clinical relapses [7]. Evidence suggests that this progression is associated with intense meningeal inflammation associated with the presence of local memory B‐cell clusters, forming an organized structure similar to lymphoid follicles (LFs) [8], probably involving recruitment of circulating CXCR5^+^ B cells induced by chemokine CXCL13 [9, 10].

LFs are highly organized structures found in secondary lymphoid organs (SLOs) containing an elevated number of different B‐cell subsets, some CD4^+^ T cells, named follicular helper T cells (*T*_FH_), and follicular dendritic cells (FDCs) [11, 12]. Lymphocyte recruitment into SLOs is thought to be driven by chemokines such as CXCL13 [9]. In this setting, following antigen exposure, interactions between *T*_FH_ cells and different B‐cell subsets, along with FDCs, support the germinal center (GC) reaction that is pivotal for the expansion of high‐affinity B‐cell clones and their subsequent differentiation into plasmablasts, plasma cells, and memory B cells [11, 12]. Although the GC reaction is vital for adequate response to different pathogens, it is also associated with pathological conditions. In autoimmunity, these ectopic LFs (eLFs) serve as a reservoir for autoreactive B‐ and T‐cell reactivation at chronic inflammation sites and are therefore a common feature of several B‐cell‐mediated autoimmune diseases such as rheumatoid arthritis (RA), systemic lupus erythematosus (SLE), and Sjögren’s syndrome [11].

In many SPMS patients, structures similar to eFLs identified below the meninges have been associated with the number and size of cortical brain lesions [12, 13]. Furthermore, the presence of eFLs also correlates with the expansion of circulating memory B cells and light‐chain neurofilament (NfL) levels in the cerebrospinal fluid (CSF) and plasma [14–16]. NfL is a neuron‐specific cytoskeletal protein that is released into the extracellular fluid following axonal injury [15]. Additionally, detection of high CXCL13 levels in the CSF of people with MS (pwMS) [16] reinforces the hypothesis of B‐cell involvement in tissue injury, neurodegeneration, and irreversible neurological impairment. Therefore, it is possible that a significant increase in different B‐cell subtypes in RRMS patients represents an early biomarker of secondary disease progression [17].

The role of B cells in MS outcomes may be related not only to antibody production but also to their ability to produce pro‐inflammatory cytokines and activate myelin‐specific CD4^+^ T cells. Indeed, high frequency of memory B cells expressing elevated surface levels of both HLA‐DR and the costimulatory molecules CD80 and CD86 [13, 18, 19], as well as IL‐6, GM‐CSF, TNF‐α, IFN‐γ and IL‐21 production have been observed in RRMS patients when compared with healthy individuals [20–25]. By contrast, circulating B cells capable of producing elevated IL‐10 levels are higher among pwMS with less disease activity than in patients with more aggressive forms of disease [26]. These data suggest that B cells play a dual role in MS.

Therefore, investigating the composition of different B‐cell subsets in RRMS patients might not only help to identify those at variable risk of disease progression but also help to develop new therapies. As such, given the expression of different biomarkers, we investigated the relationship between the frequencies of circulating transitional, naïve and memory B cells, as well as plasmablasts, and disease activity, evaluated here by occurrence of clinical relapses and severity of neurological impairment. We also correlated B‐cell subsets with plasma levels of CXCL13 and NfL.

## 2. Methods

### 2.1. Patients

For our study, 45 patients in clinical remission, with a definite diagnosis of relapsing–remitting MS, according to the McDonald 2010 criteria [27], were recruited from January 2022 to January 2024 from Gaffrée e Guinle University Hospital/UNIRIO (Rio de Janeiro, Brazil). Demographic data such as gender and age at disease onset were obtained from medical records (Table 1). Patients in clinical remission were recruited from Gaffrée e Guinle Hospital (HUGG/UNIRIO) (Table 1). Patients with additional autoimmune diseases, neoplasms, pregnant women, smokers, or users of illicit substances were excluded. Patients treated with corticosteroids within an interval of less than 3 months before the day of blood sampling were excluded. Patients using medications that deplete B cells (rituximab and ocrelizumab) were also excluded. The neurological disability status of patients was evaluated by one of the authors (CCV) and was scored according to the Expanded Disability Status Scale (EDSS) [28]. Information about the occurrence of clinical relapses subsequent to blood sampling for immunology over the 1‐year follow‐up was obtained from medical records. A clinical relapse is defined as a new or worsening neurological symptom lasting at least 24 h, in the absence of fever or infection, and separated by at least 30 days from the prior relapse. As control, 20 healthy individuals, matched by age and gender, were recruited to participate in the study. With regard to ancestry, we classified as Afrodescendant those with African descent until the third generation. After a complete description of the study to the participants, written informed consent was obtained from each individual. The study was approved by the Ethics Committee for Research on Human Subjects of the Federal University of the State of Rio de Janeiro (UNIRIO) (CAAE number: 43009015.6.0000.5258).

**Table 1:** Data from the control group and stable and unstable RRMS patients.

Group data	Control^a^	Stable MS^b^	Unstable MS^c^
Gender, female/male (*n*)	15/5	24/6	12/3
Age [(years), mean ± SD]^d^	41.8 ± 13.7	37.6 ± 10.3	46.5 ± 9.36
Disease duration [(years), mean ± SD]^e^	NA	11.5 ± 8.5	14 ± 9.1
Ethnicity			
Caucasian	9	13	4
Afro descendent	11	17	11
EDSS [median (range)]^f^			
t0	NA	1.5 ± 1.2	2.5 ± 1.3
t1	NA	1.5 ± 1.2	3.0 ± 0.9
CXCL13 (pg/mL)^h^	30 ± 33.1^∗#^	234 ± 221.8^∗^^ƒ^	471.3 ± 290.4^#ƒ^
NfL (pg/mL)^i^	5.4 ± 6.5^∗^	22 ± 27.2^#^	51.4 ± 32^∗#^
DMT^g^			
Fingolimod	NA	7	3
Dimethylfumarate	NA	6	4
Natalizumab	NA	7	3
IFN‐β	NA	5	3
Glatiramer acetate	NA	5	2

*Note:* Data from ^a^healthy individuals (control) and relapsing‐remitting multiple sclerosis who ^b^relapsed (Unstable) or ^c^not (Stable) during 1 year follow up. ^d^Age (years) refers to age when the blood samples were collected. ^e^Disease duration refers to the number of years since disease onset. ^f^EDSS, Expanded Disability Status Scale, evaluated just before (t0) and 1 year after (t1) blood sampling, and ^g^the treatment time in years with disease‐modifying therapy (DMT). ^h,i^Plasma levels of CXCL13 and NfL in healthy subjects (control) and pwMS [stable (*n* = 30) and relapsed (unstable, *n* = 15)] were quantified using ELISA. The mean values of the three subgroups were compared (one‐way ANOVA). CXCL13  ^∗,#^ *p* < 0.0001 and ^ƒ^ *p* = 0.0411. NfL:  ^∗^ *p* < 0.0008 and ^#^ *p* = 0.079 (Kruskal–Wallis followed by Dunn’s test. Multivariate Firth logistic regression models and ROC analysis of Firth Logistic Regression Models were also applied).

Abbreviation: NA, not applicable.

### 2.2. Plasma and Cell Cultures

Peripheral blood was collected from participants in heparin‐containing tubes, and both plasma and mononuclear cells (PBMC) were obtained by centrifugation on the Ficoll–Hypaque density gradient. For some experiments, whole B cells were isolated from PBMC using EasySep Human Pan‐B‐Cell Enrichment Kit (StemCell Technologies). After counting cells in trypan blue solution (10% v/v), viable PBMCs (1 × 10^6^/mL) or B cells (1 × 10^4^/mL) were suspended in RPMI‐1640 medium supplemented with 2 μM of L‐glutamine (GIBCO, Carlsbad, CA, USA), 10% fetal calf serum (FCS), 20 U/mL of penicillin, 20 μg/mL of streptomycin, and 20 mM of HEPES buffer. In order to analyze stimulation‐induced IL‐17 and IL‐10 response, PBMC or B‐cell cultures were stimulated for 4 h with phorbol 12‐myristate 13‐acetate (PMA, 20 ng/mL; Sigma–Aldrich) plus ionomycin (600 ng/mL; Sigma–Aldrich). For cytometry analysis of cytokine‐producing B‐cell subsets, brefeldin A (10 μg/mL; Sigma–Aldrich) was also added to PBMC cultures. The cell cultures were maintained at 37°C in a humidified 5% CO_2_ incubator for incubation.

### 2.3. Flow Cytometry Analysis

To determine the frequency of different B lymphocyte subtypes, PBMCs were labeled with monoclonal antibodies (mAbs) directed against different markers [anti‐CD19‐Super Bright 600 (clone SJ25C1); anti‐IgD‐PerCP‐eFluor 710 (clone IA62); anti‐CD38‐PE (clone HIT2); anti‐CD27‐Super Bright 436 (clone 0353); anti‐HLA‐DR‐APC‐eFluor780 (clone LN3); anti‐IL‐10‐AlexaFluor 488 (clone JES39DT), anti‐IL‐17A‐APC (eBio64DEC17), anti‐CD138‐PECy‐7 (clone MI15)]. These mAb combinations allowed us to determine the following B cells: total (CD19^+^), naïve (CD38^+^ CD27^−^CD19^+^), transitional (CD27^−^CD38^++^ CD19^+^), memory subsets (CD27^−^CD38^−^CD19^+^, CD27^+^CD38^+^ CD19^+^, CD27^+^CD38^−^ CD19^+^), and total and CD138^+^ plasmablast (CD38^++^CD27^++^ CD19^+^). These cells were also analyzed for HLA‐DR and IgD expression, as well as synthesis of IL‐17 and IL‐10. All monoclonals were purchased from BioLegend (San Diego, CA, USA), eBioscience (Waltham, Massachusetts, USA), and BD Bioscience (San Diego, CA, USA). Briefly, the PBMCs were incubated with different mAb combinations for surface markers (anti‐CD19, anti‐CD27, anti‐CD38, anti‐IgD, anti‐HLA‐DR, and anti‐CD138) at room temperature in the dark, according to manufacturer’s instructions. After 30 min, the cells were washed with PBS + 2%FBS at room temperature before cell permeabilization, which was performed by incubating cells at 4°C for 20 min with Cytofx/Cytoperm solution (BD Pharmigen, San Diego, CA). After washing, mAbs against IL‐17 and IL‐10 were added and incubated for a further 30 min at 4°C. The cells were acquired on Attune NxT flow cytometers (Thermo Fisher Corporation) and analyzed using FlowJo. Isotype control antibodies and single‐stained samples were used to periodically check the settings and gates on the flow cytometer. After acquisition of 100,000–200,000 events, lymphocytes were gated based on forward and side scatter properties after the exclusion of dead cells using propidium iodide [PI + cells mean: 2.13% (range 0.78%–3.1%)] and doublets (Supporting Information 1: Figure S1).

### 2.4. ELISA Assay

CXCL13 levels in plasma were quantified using the ELISA technique with Thermo Scientific Human CXCL13 (BCL) kits following manufacturer’s instructions (Thermo Fischer–Waltham, Massachusetts, USA). The quantification of plasma NfL levels was performed using the ELISA technique (Enzyme‐Linked Immunosorbent Assay) with Elabscience Human NEFL ELISA kits (NfL, Light Polypeptide) following manufacturer’s instructions. Recombinant human CXCL13 (1.37–1000 pg/mL) and NfL (3.13–200 pg/mL) were employed to construct standard curves. IL‐17 and IL‐10 levels were determined using ELISA with Human IL‐17 (KHC0101) and IL‐10 (KAC1591) ELISA Kits (Thermo Fischer‐ Invitrogen) following manufacturer’s instructions. Recombinant human IL‐17 and IL‐10, at concentrations ranging from 15 to 1000 pg/mL (IL‐17) and 7.8 to 500 pg/mL (IL‐10), were used to construct standard curves. In those assays, the reaction was revealed with streptavidin‐horseradish peroxidase using 3.3′, 5.5′‐tetramethyl‐benzidine (TMB) as a substrate. The plates were read at 450 nm and 550 nm in an ELISA reader.

### 2.5. Statistical Analysis

The statistical analysis was carried out using Prism 8.0 software (GraphPad Software). Comparisons between immune assays from the two patient subgroups were performed by using Kruskal–Wallis followed by Dunn’s test. Additionally, the results were corrected by Bonferroni. The nonparametric Mann–Whitney *U* test and Student’s *t*‐test were applied to determine whether the two groups were statistically different for nonparametric and parametric variables, respectively. Multivariate Firth logistic regression models were applied to evaluate the relationship between B‐cell subsets and CXCL13 levels with a binary outcome variable (relapse/no relapse) taking into account multiple confounding variables simultaneously (age, EDSS, gender, therapy). ROC analysis of Firth logistic regression models was performed. To evaluate the robustness of the predictive linear models, leave‐one‐out cross‐validation (LOOCV) was performed. In each iteration, one participant was excluded from the dataset, and the Firth logistic regression model was refitted using the remaining participants. The fitted model was used to estimate the predicted probability for the excluded participant. This process was repeated until every participant had served as the test observation once. The validated predicted probabilities were then combined to construct ROC curves. The optimal cutoff value was determined using Youden’s index, and the corresponding sensitivity, specificity, and accuracy were calculated. Correlations between nonparametric variables were investigated using Spearman’s correlations. Aiming to evaluate the strength and direction of association between two ranked or ordinal variables, Spearman’s rank correlation coefficient with 95% confidence intervals was estimated using the percentile bootstrap method with 2000 resamples. Significance for all experiments was *p* < 0.05.

## 3. Results

### 3.1. Circulating B‐Cell Subsets are Correlated With Severity of Neurological Impairment in MS

For the present study, different B‐cell subsets in the peripheral blood from healthy individuals (control, *n* = 20) and pwMS in remission phase (*n* = 45) were collected (Table 1). According to data regarding the occurrence of relapses over the 1‐year follow‐up period, pwMS were grouped in Table 1 as either clinically stable (stable, *n* = 30) or relapsed (unstable, *n* = 15). For ethnicity, the majority is Afrodescendant, mainly among unstable group (73%). Neurological impairment, determined by EDSS scale, was evaluated just before (t0) and 1 year after (t1) blood sampling. For DMT, stable pwMS were undergoing treatment with fingolimod (FGL, *n* = 7), dimethyl fumarate (DMF, *n* = 6), natalizumab (NATz, *n* = 7), interferon (IFN, *n* = 5), and glatiramer acetate (AG, *n* = 5). During the 1‐year follow‐up period, 15 pwMS undergoing DMT [FGL (*n* = 3), DMF (*n* = 4), NATz (*n* = 3), IFN (*n* = 3), and AG (*n* = 2)] showed one or more clinical relapses (Table 1). With regard to plasma CXCL13, higher levels of this chemokine were observed in pwMS samples, mainly among unstable patients (Table 1). Furthermore, the plasma NfL concentration was also significantly higher in relapsed group as compared with the control one. Regarding treatment, no significant difference was observed between DMT regimes and B‐cell subsets in either patient subgroup (Supporting Information 2: Figure S2). Moreover, since our experiment was performed with MS patients with an unbalanced DMT regimen, analysis by using a multivariate logistic regression demonstrated that B‐cell subsets by treatment are not different. These variables presented wide confidence intervals and high standard errors, likely reflecting the limited sample size and sparse observations within treatment subgroups.

Taking into account the different B‐cell subsets shown in Supporting Information 1: Figure S1, pwMS (*n* = 45) presented higher frequency of total and CD138^+^ and HLA‐DR^+^IgD^−^ plasmablasts, HLA‐DR^+^IgD^+^ and HLA‐DR^+^IgD^−^ transitional cells, and different memory B‐cell subsets (HLA‐DR^+^IgD^+^CD27^+^ CD38^+^, HLA‐DR^+^IgD^−^CD27^+^CD38^+^ and HLA‐DR^+^IgD^+^ CD27^+^CD38^−^) when compared with healthy individuals (Table 2). Also, elevated percentages of HLA‐DR^−^IgD^+^ memory B cells were observed in the MS group. By contrast, the frequency of HLA‐DR^−^IgD^+^ cells among transitional and plasmablasts was lower in patients than in the control (Table 2).

**Table 2:** The frequency of different B‐cell subsets in healthy subjects and RRMS patients.

Cells (%, mean ± SD)	Control	RRMS	*p*‐Value
Total	6.8 ± 2.4	9.5 ± 5.8	0.1650
HLA‐DR^+^ IgD^+^	41.1 ± 7.7	53.1 ± 22.8	0.1317
HLA‐DR^+^ IgD^−^	14.1 ± 6	15.5 ± 10.6	0.1370
HLA‐DR^−^ IgD^+^	21.8 ± 8.8	28.2 ± 25.4	0.5146
IL17^+^IL10^−^	36.1 ± 16.5	9.3 ± 4.7	**<0.0001**
IL17^−^IL10^+^	3.1 ± 2	4.1 ± 2.8	0.2122
IL17^+^IL10^+^	1.2 ± 1.1	0.5 ± 0.8	0.1334
Transitional	7.1 ± 2.6	11.5 ± 10.1	0.2646.
HLA‐DR^+^ IgD^+^	30.9 ± 17.3	68.8 ± 27.1	**0.0011**
HLA‐DR^+^ IgD^−^	1.6 ± 2.2	10.8 ± 7.1	**<0.0001**
HLA‐DR^−^ IgD^+^	52.4 ± 19	17.3 ± 14.6	**<0.0001**
IL17^+^IL10^−^	5.4 ± 2.3	5.9 ± 4.1	0.9677
IL17^−^IL10^+^	5 ± 1.9	5.7 ± 4.3	0.6981
IL17^+^IL10^+^	0.4 ± 0.6	0.5 ± 0.7	0.8098
Naïve	36.7 ± 12.5	32.5 ± 12.2	0.4243
HLA‐DR^+^ IgD^+^	73.9 ± 8.3	68.6 ± 26.1	0.5303
HLA‐DR^+^ IgD^−^	18.1 ± 6.7	14.9 ± 10.5	0.4574
HLA‐DR^−^ IgD^+^	10.5 ± 1.2	12.7 ± 10.8	0.3111
IL17^+^IL10^−^	55 ± 23	5.5 ± 3.2	**<0.0001**
IL17^−^IL10^+^	3 ± 1.8	5.7 ± 2.9	0.0664
IL17^+^IL10^+^	2.4 ± 1.9	0.7 ± 0.8	**0.0012**
Plasmablast	1.1 ± 0.9	2.9 ± 1.1	**0.0020**
CD138^+^	7.9 ± 5	18.2 ± 9.7	**0.0096**
HLA‐DR^+^ IgD^+^	10.2 ± 7.6	14 ± 11.8	0.3144
HLA‐DR^+^ IgD^−^	0.6 ± 0.7	9.7 ± 9.1	**0.0116**
HLA‐DR^−^ IgD^+^	67 ± 29	28.1 ± 25.4	**0.0004**
IL17^+^IL10^−^	9.9 ± 2.3	6.1 ± 5.3	**0.0454**
IL17^−^IL10^+^	6.3 ± 4.2	4.9 ± 3.7	0.1211
IL17^+^IL10^+^	4.1 ± 2.5	0.9 ± 1	**<0.0001**
Memory			
CD27^−^CD38^−^	16.6 ± 5.7	21.1 ± 0.7	0.0642.
HLA‐DR^+^ IgD^+^	48.5 ± 7.4	52.1 ± 16.5	0.5106
HLA‐DR^+^ IgD^−^	31.5 ± 8.8	36.4 ± 17	0.4043
HLA‐DR^−^ IgD^+^	0.6 ± 0.4	16.2 ± 14.1	**0.0027**
IL17^+^IL10^−^	53 ± 15.2	4.2 ± 3.3	**<0.0001**
IL17^−^IL10^+^	6 ± 2.2	5.7 ± 4.1	0.1748
IL17^+^IL10^+^	2.9 ± 1.7	1.2 ± 1	0.2876
CD27^+^CD38^+^	8.3 ± 5.3	11.5 ± 7.4	0.1449
HLA‐DR^+^ IgD^+^	6.1 ± 3.2	44.3 ± 16.3	**<0.0001**
HLA‐DR^+^ IgD^−^	13.9 ± 9.9	29.1 ± 18.7	**0.0150**
HLA‐DR^−^ IgD^+^	2.3 ± 1.8	18.4 ± 11.3	**0.0016**
IL17^+^IL10^−^	10.3 ± 4	5.1 ± 3.6	**<0.0001**
IL17^−^IL10^+^	8.6 ± 3.7	7.1 ± 3.1	0.2968
IL17^+^IL10^+^	3.3 ± 1.4	1.7 ± 1.5	0.1017
CD27^+^CD38^−^	20 ± 7.3	18.3 ± 10.1	0.3011
HLA‐DR^+^ IgD^+^	5.8 ± 2.2	24.3 ± 10.9	**<0.0001**
HLA‐DR^+^ IgD^−^	21.7 ± 6.4	22.4 ± 18.4	0.1624
HLA‐DR^−^ IgD^+^	2.9 ± 2.2	22.5 ± 17.9	**0.0021**
IL17^+^IL10^−^	38.2 ± 21.4	6.6 ± 5.6	**<0.0001**
IL17^−^IL10^+^	9.5 ± 4.8	3.3 ± 3.1	0.1088
IL17^+^IL10^+^	1.3 ± 1	0.8 ± 1	0.3981

*Note:* The percentage of total (CD19^+^) and different B subsets [Transitional (CD27^−^CD38^++^), naïve (CD27^−^CD38^+^), total and CD138^+^ plasmablasts (CD27^++^CD38^++^) and memory cells (CD27^−^CD38^−^; CD27^+^CD38^+^ and CD27^+^CD38^−^)] was determined by flow cytometry in healthy subjects (control, *n* = 20) and RRMS patients (*n* = 45). Significance was calculated by comparing control and MS patients by using Mann–Whitney *U* test (significance *p* < 0.05). Values in bold statistically significant differences between the groups (*p* < 0.05)

Cytokines such as IL‐17 and IL‐10 have been classically associated with the promotion and suppression of inflammation in MS [29]. Taking into account the strategy of IL‐17^+^ and/or IL‐10^+^ B‐cell identification in Supporting Information 1: Figure S1, Table 2 shows that a higher proportion of IL‐17^+^IL‐10^−^ in total, naïve, plasmablast, and all memory B‐cell subsets was observed in the control group than in MS samples. Moreover, an elevated percentage of IL‐17^+^IL‐10^+^ plasmablasts and naïve cells was also seen in the healthy group (Table 2).

Regarding the severity of neurological impairment and RRMS‐derived B cells, different plasmablast subsets (CD138^+^, HLA‐DR^+^ IgD^−^, and IL‐17^+^IL‐10^−^) correlated with EDSS score. HLA‐DR^+^ IgD^−^ and IL‐17^+^IL‐10^−^ memory CD27^+^CD38^+^ B cells also positively correlated with severity of neurological impairment (Table 3). By contrast, the proportion of HLA‐DR^−^IgD^+^ and IL‐17^−^IL‐10^+^ among transitional and IL‐17^−^IL‐10^+^ plasmablasts was observed in patients with lower neurological impairment (Table 3).

**Table 3:** Correlation between B‐cell subsets and neurological disability in MS patients.

B cell subsets	EDSS	
	*R* ^a^	*p*‐Value
Total	0.1213	0.4274
HLA‐DR^+^ IgD^+^	0.1362	0.3724
HLA‐DR^+^ IgD^−^	0.2587	0.500
HLA‐DR^−^ IgD^+^	0.1237	0.4237
IL17^+^IL10^−^	0.1601	0.2991
IL17^−^IL10^+^	−0.1577	0.3009
IL17^+^IL10^+^	0.1551	0.3146
Transitional	0.1282	0.4068
HLA‐DR^+^ IgD^+^	−0.2589	0.0860
HLA‐DR^+^ IgD^−^	0.2091	0.1681
HLA‐DR^−^ IgD^+^	**−0.4136**	**0.0047**
IL17^+^IL10^−^	0.0199	0.8975
IL17^−^IL10^+^	**−0.4498**	**0.0022**
IL17^+^IL10^+^	0.2040	0.1842
Naïve	0.0854	0.5815
HLA‐DR^+^ IgD^+^	−0.1076	0.4816
HLA‐DR^+^ IgD^−^	0.1109	0.4309
HLA‐DR^−^ IgD^+^	−0.1615	0.2891
IL17^+^IL10^−^	0.2711	0.8613
IL17^−^IL10^+^	−0.0414	0.7920
IL17^+^IL10^+^	−0.0205	0.8947
Plasmablast	0.1658	0.2820
CD138^+^	**0.6639**	**<0.0001**
HLA‐DR^+^ IgD^+^	0.2659	0.0810
HLA‐DR^+^ IgD^−^	**0.5448**	**0.0001**
HLA‐DR^−^ IgD^+^	−0.2511	0.0918
IL17^+^IL10^−^	**0.3217**	**0.0312**
IL17^−^IL10^+^	**−0.3033**	**0.0429**
IL17^+^IL10^+^	−0.1144	0.9412
Memory		
CD27^−^CD38^−^	0.2178	0.1507
HLA‐DR^+^ IgD^+^	0.1761	0.3761
HLA‐DR^+^ IgD^−^	0.2317	0.0611
HLA‐DR^−^ IgD^+^	0.1277	0.4051
IL17^+^IL10^−^	0.1213	0.4274
IL17^−^IL10^+^	−0.1866	0.2456
IL17^+^IL10^+^	0.1655	0.2815
CD27^+^CD38^+^	0.1846	0.2303
HLA‐DR^+^IgD^+^	0.1282	0.4068
HLA‐DR^+^IgD^−^	**0.3121**	**0.0312**
HLA‐DR^−^ IgD^+^	0.2321	0.1079
IL17^+^IL10^−^	**0.5056**	**0.0004**
IL17^−^IL10^+^	−0.1233	0.4228
IL17^+^IL10^+^	−0.1409	0.3381
CD27^+^CD38^−^	0.1035	0.4986
HLA‐DR^+^ IgD^+^	0.1317	0.5141
HLA‐DR^+^ IgD^−^	0.2651	0.0855
HLA‐DR^−^ IgD^+^	−0.1288	0.4850
IL17^+^IL10^−^	0.1655	0.2818
IL17^−^IL10^+^	−0.1091	0.5644
IL17^+^IL10^+^	−0.1067	0.5477

*Note:* The frequency of different B‐cell subtypes (CD19^+^) [Transitional (CD27^−^CD38^++^), naïve (CD27^−^CD38^+^), total and CD138^+^ plasmablast (CD27^++^CD38^++^), and memory (CD27^−^CD38^−^; CD27^+^CD38^+^ and CD27^+^CD38^−^)] cells was evaluated in pwMS by flow cytometry. Furthermore, the differential expression of other surface markers (HLA‐DR and IgD) and cytokines (IL‐10/IL‐17) was analyzed by flow cytometry. All those B cell subsets were correlated with neurological impairment, determined by EDSS score. *ρ* = Spearman’s rank correlation coefficient. Significance *p* < 0.05. Statistically significant *p*‐values (*p* < 0.05) are highlighted in the bold.

^a^95% confidence intervals were estimated using the percentile bootstrap method with 2000 resamples.

### 3.2. B‐Cell Subset Imbalance Was Associated With Clinical Activity of MS

Given that RRMS is characterized by unpredictable relapses followed by remission periods, our next objective was to determine if the frequency of different RRMS‐derived B‐cell subsets during remission could be associated with the occurrence of clinical relapses during the 1‐year follow‐up even when undergoing DMT.

**Figure 1:**
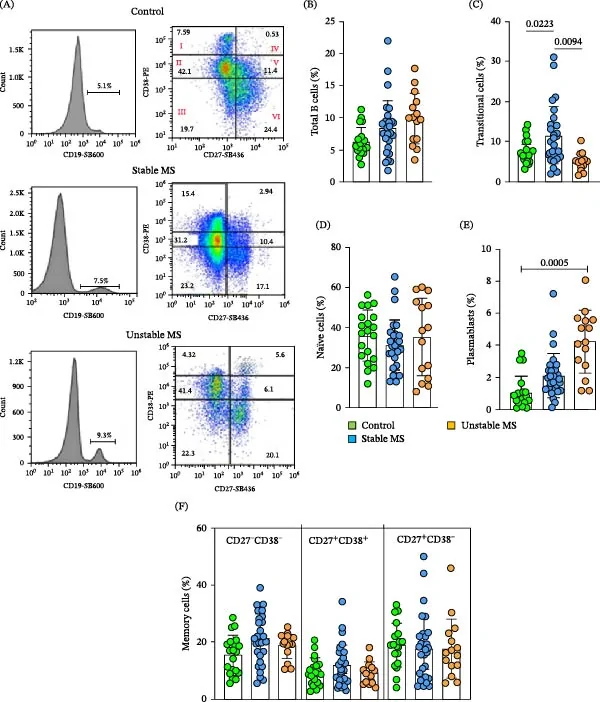
Identification of B‐cell subsets according to CD38 and CD27 expression in stable and unstable RRMS patients. Assuming the gating strategy shown in (A) (total; I‐ transitional; II‐ naïve; III‐ memory CD27^−^; IV‐ plasmablast; V‐ memory CD27^+^CD38^+^; VI‐ memory CD27^+^CD38^−^), the percentage of (B) Total (CD19^+^ cells), (C) transitional (CD19^+^CD27^−^CD38^++^), (D) naive (CD19^+^CD27^−^CD38^+^), (E) plasmablast (CD27^++^CD38^++^), and (F) different memory B‐cell subsets (CD27^−^CD38^−^, CD27^+^CD38^+^, CD27^−^CD38^+^) was evaluated in healthy individuals (control, *n* = 20) and RRMS patients grouped according to occurrence of relapses during the 1‐year follow‐up [stable (*n* = 30) and unstable (*n* = 15) patients]. (B)–(F) shows data as mean ± SD of nine independent experiments with three to six samples per experiment. Significance was calculated by comparing control and MS subgroups using one‐way ANOVA, followed by Tukey post hoc test. Multivariate Firth logistic regression models and ROC analysis of Firth logistic regression models were also applied. The *p*‐values are indicated in the figure.

Assuming the gating strategy shown in Figure 1A, the frequency of transitional cells was higher in stable patients throughout the 1‐year follow‐up period by comparison with the relapsed (unstable) groups (Figure 1C). On the other hand, the percentage of plasmablasts was higher among unstable pwMS (Figure 1E), with no difference for the other B‐cell subsets.

**Figure 2:**
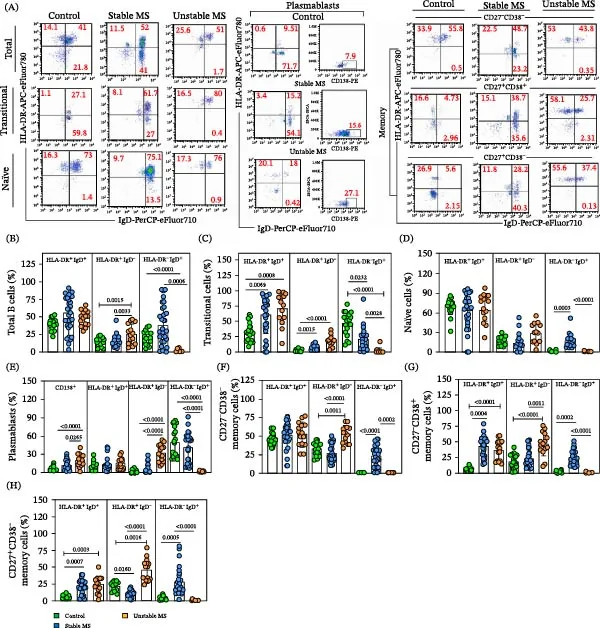
Evaluation of B‐cell subsets according to HLA and IgD expression in stable and unstable RRMS patients. Adopting the gating strategy shown in (A), circulating B‐cell subsets were phenotypically identified as: (B) total (CD19^+^), (C) transitional (CD19^+^CD27^−^CD38^++^), (D) naive (CD19^+^ CD27^−^CD38^+^), (E) plasmablast (CD27^++^CD38^++^), and different memory B‐cell (CD19^+^) subsets [(F) (CD27^−^CD38^−^), (G) (CD27^+^CD38^+^), and (H) (CD27^+^CD38^−^)], according to their expression of HLA‐DR and IgD. Data are shown as mean ± SD of nine independent experiments with three to six samples per experiment. Significance was calculated using Kruskal–Wallis followed by Dunn’s test, comparing control (*n* = 20) and pwMS, grouped according to occurrence of relapses during the 1‐year follow‐up [stable (*n* = 30) and unstable (*n* = 15) patients]. Multivariate Firth logistic regression models and ROC analysis of Firth logistic regression models were also applied. The *p*‐values are indicated in the figure.

Regarding HLA‐DR and IgD expression, and assuming the gating strategy shown in Figure 2A, the percentage of HLA‐DR^+^IgD^+^ and HLA‐DR^+^IgD^−^ transitional (Figure 2C) and HLA‐DR^+^IgD^+^ memory B‐cell subsets [CD27^+^CD38^+^ (Figure 2G) and CD27^+^CD38^−^ (Figure 2H)] was significantly higher in both MS subgroups by comparison with the control, with no difference observed between stable and unstable patients. The occurrence of relapses was mainly observed in pwMS who showed higher frequency of total (Figure 2B), plasmablast (Figure 2E), and all memory B‐cell subsets (Figure 2F–H) with HLA‐DR^+^IgD^−^ phenotype at baseline. Furthermore, increased percentages of CD138^+^ plasmablasts at t0 were associated with clinical relapse during follow‐up (Figure 2E). By contrast, the presence of HLA‐DR^−^IgD^+^ among naïve (Figure 2D) and all memory B cells (Figure 2F–H) was higher in stable pwMS than in either control or relapsed patients. Moreover, total (Figure 2B), transitional (Figure 2C), and plasmablasts negative for HLA‐DR but positive for IgD were higher in stable patients by comparison with unstable ones.

**Figure 3:**
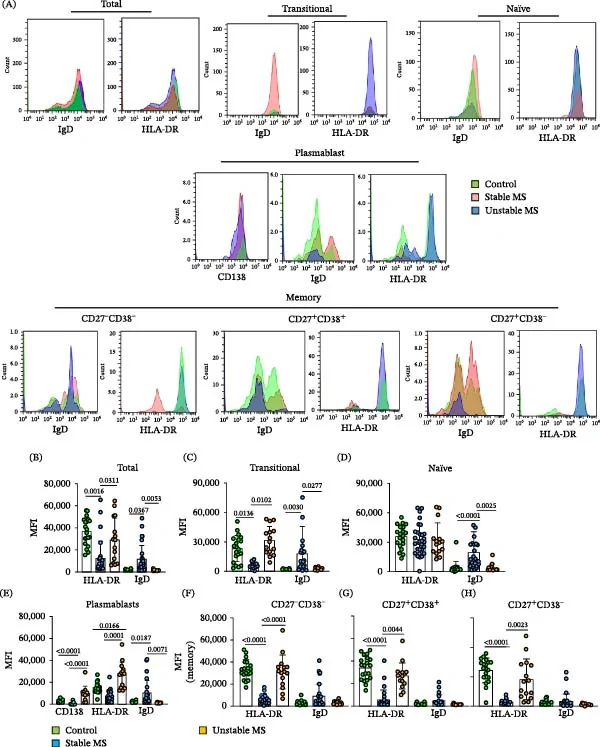
Intensity of HLA‐DR and IgD expression by B‐cell subset in MS patients according to relapses. (A) The mean fluorescence intensity (MFI) of HLA‐DR and IgD markers per (B) total (CD19^+^), (C) transitional (CD27^−^CD38^++^), (D) naïve (CD27^−^CD38^+^), (E) plasmablast (CD27^++^CD38^++^), and different memory B‐cell subsets [(F) (CD27^−^CD38^−^), (G) (CD27^+^CD38^+^) and (H) (CD27^+^CD38^−^)] analyzed and compared in the control (*n* = 20) and stable (*n* = 30) and relapsed (unstable, *n* = 15) pwMS. Data are shown as mean ± SD of nine independent experiments with three to six samples per experiment. Significance was calculated by comparing control and MS patient subgroups using Kruskal–Wallis followed by Dunn’s test. Multivariate Firth logistic regression models and ROC analysis of Firth logistic regression models were also applied. The *p*‐values are indicated in the figure.

Regarding mean fluorescence intensity (MFI), as shown in Figure 3A, lower intensity of HLA‐DR per total (Figure 3B), transitional (Figure 3C), plasmablast (Figure 3E), and all memory B‐cell subsets (Figure 3F–H) was observed in samples from clinically stable patients, by comparison with control and unstable groups. Interestingly, total (Figure 3B), transitional (Figure 3C), naïve (Figure 3D), and plasmablast (Figure 3E) cells expressing high IgD levels were mainly observed in patients without relapse during the 1‐year follow‐up.

Regarding cytokines, and assuming the gating strategy shown in Figure 4A, the frequency of IL‐17^+^IL‐10^−^ phenotype among total (Figure 4B), naïve (Figure 4D), and memory B‐cell subsets [CD27^−^CD38^−^ (Figure 4F) and CD27^+^CD38^−^ (Figure 4H) was significantly higher in the control group than pwMS. Also, a lower percentage of memory IL‐17^+^IL‐10^−^CD27^+^CD38^+^ B cells was observed in stable patients as compared with the control group (Figure 4G). At baseline, a higher proportion of IL‐17^+^IL‐10^−^ phenotype in the transitional (Figure 4C), naïve (Figure 4D), plasmablast (Figure 4E), and memory B‐cell subsets [CD27^−^CD38^−^ (Figure 4F) and CD27^+^CD38^+^ (Figure 4G)] was associated with occurrence of relapses over time. By contrast, a higher percentage of total (Figure 4B), transitional (Figure 4C) and plasmablasts (Figure 4E) with IL‐17^−^IL‐10^+^ phenotype was seen in stable patients than in relapsed ones. The proportion of IL‐17^+^IL‐10^+^ plasmablasts (Figure 4E) and IL‐17^+^IL‐10^+^ memory B‐cell subsets [CD27^−^CD38^−^ (Figure 4F) and CD27^+^CD38^+^ (Figure 4G)] was elevated in the control group. In B‐cell cultures, the levels of IL‐17 released in the control samples were significantly higher than for stable and relapsed MS patients, with no difference for IL‐10 release (Supporting Information 3: Figure S3). On the other hand, among pwMS, while IL‐17 release was greater, the release of IL‐10 was lower in unstable B‐cell cultures (Supporting Information 3: Figure S3). Like IL‐10^+^ and IL‐17^+^‐secreting B cells, the levels of those cytokines released by total B cells were not correlated with disease activity (data not shown).

Figure 4Comparison between different cytokine‐producing B‐cell subsets and clinical activity of MS. PBMC cultures (1 × 10^6^/mL) from healthy control (Control, *n* = 20) and stable (*n* = 30) and unstable (*n* = 15) RRMS were stimulated with PMA and ionomycin for 4 h, and the frequency of (B) total, (C) transitional, (D) naïve, (E) plasmablasts, and different memory B‐cell subsets [(F) (CD27^−^CD38^−^), (G) (CD27^+^CD38^+^), and (H) (CD27^+^CD38^−^)] capable of producing IL‐17 and/or IL‐10 was determined adopting the gating strategy shown in (A). Data are shown as mean ± SD of nine independent experiments with three to six samples per experiment. Significance was calculated by comparing control and MS patient subgroups using Kruskal–Wallis followed by Dunn’s test. Multivariate Firth logistic regression models and ROC analysis of Firth logistic regression models were also applied. The *p*‐values are indicated in the figure.

**Figure:**
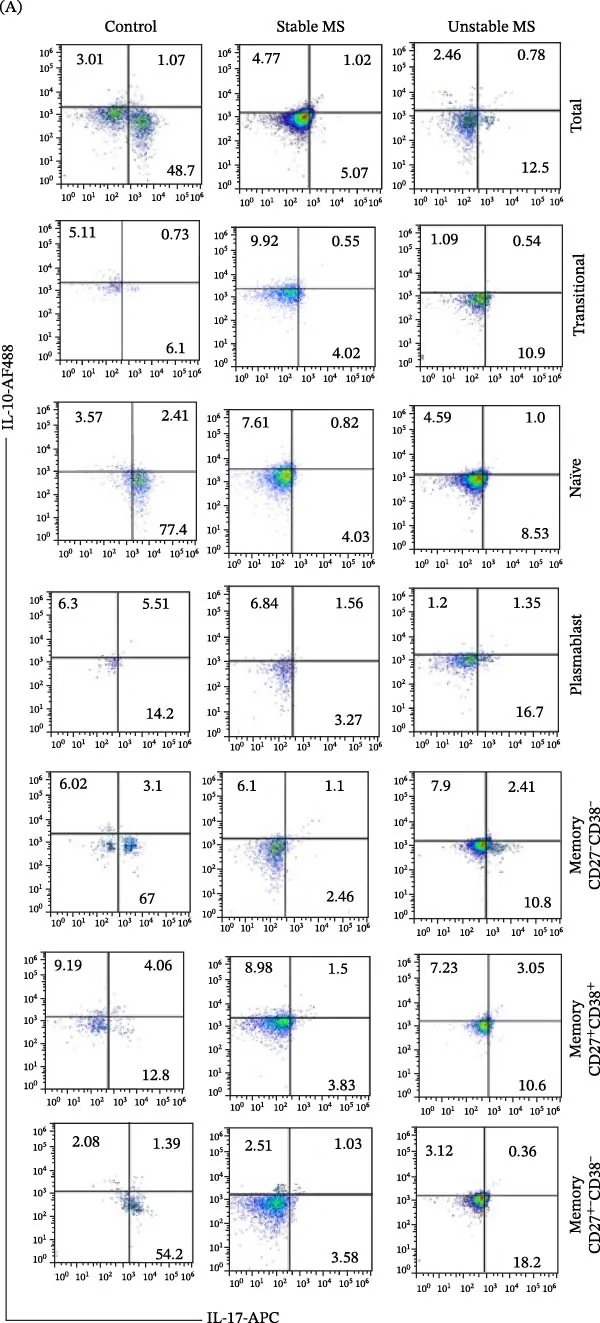

**Figure:**
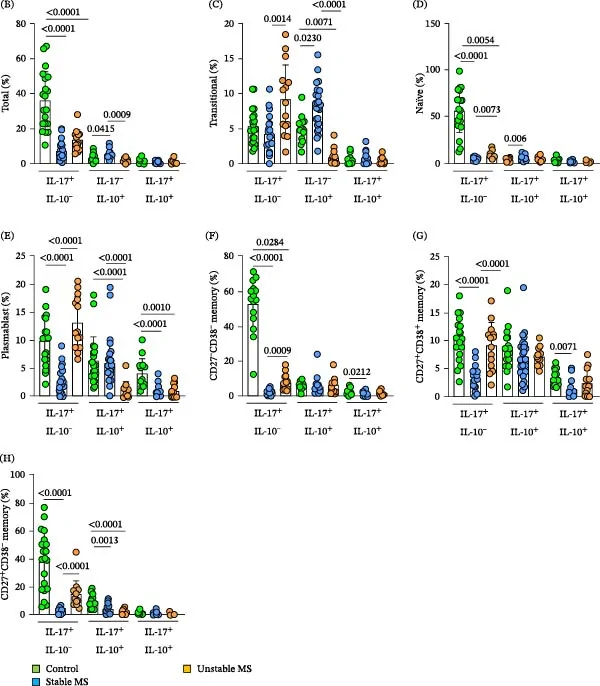

Applying Multivariate logistic regression adjusted for the same control variables (age, EDSS, and gender) (Supporting Information 4: Table S1), the plasma levels of CXCL13, the percentage of CD138^+^ plasmablasts, and the frequency of HLA‐DR^+^IgD^−^ cells among naïve, plasmablasts, transitional, and memory B cells (CD27^−^CD38^−^ and CD27^+^CD38^−^) remained significantly higher among unstable patients. Moreover, the intensity of HLA‐DR expression per plasmablast and memory B‐cell subsets and MFI of CD138 on plasmablasts remained higher among B cells from unstable patients, even after adjusting for the same control variables. On other hand, the intensity of IgD expression persisted significantly higher among naïve, plasmablast, and CD27^+^CD38^+^ memory B cells from stable MS patients. Concerning the cytokine profile, even after adjusting for age, EDSS, and gender, the percentage of transitional and memory B‐cell subsets (CD27^−^CD38^−^ and CD27^+^CD38^−^) with IL‐17^+^IL‐10^−^ phenotype remained significantly higher in relapsed group, while IL‐17^−^IL‐10^+^ plasmablast frequency remained higher in stable patients.

Finally, by applying both ROC analysis of Firth Logistic Regression Models (Supporting Information 5: Table S2) and internal validation using LOOCV (Supporting Information 6: Table S3), the following parameters showed an excellent discriminatory performance for predicting disease stability or clinical relapses in 1‐year follow‐up: percentages of HLA‐DR^−^IgD^+^ naïve, plasmablast, and memory B cells, IL‐17^−^IL‐10^+^ transitional cells, IL‐17^+^IL‐10^−^ plasmablasts and memory B cells, as well as the intensity of expression of IgD per transitional cell and of CD138 per plasmablast. The LOOCV‐AUC, sensitivity, specificity, and overall accuracy were above 90% (Supporting Information 7: Table S4).

### 3.3. Plasma Levels of CXCL13 and NfL Were Correlated With Different B‐Cell Subsets in MS Patients

It is known that the migration of B cells expressing CXCR5 to LFs is mediated by the chemokine CXCL13 [9]. As demonstrated in Supporting Information 8: Figure S4, most circulating B cells express CXCR5, with no difference between the three different subgroups. When compared with the control group, the plasma levels of CXCL13 were higher in pwMS samples, mainly among unstable patients (Table 1). Furthermore, the plasma NfL concentration, a biomarker of MS activity [14], presented a tendency to increase in relapsed pwMS (Table 1, *p* = 0.079). The mean NfL levels in the control group were very low (most of them below the detection limit). Among stable and relapsed pwMS, CXCL13 levels directly correlated with the frequency of total and CD138^+^ plasmablasts and memory B cells with phenotype HLA‐DR^+^IgD^−^ (Table 4). Regarding NfL, plasma levels of this protein, in both MS subgroups, positively correlated with CD138^+^ and HLA‐DR^+^IgD^−^ plasmablasts but negatively correlated with HLA‐DR^−^IgD^+^ cell phenotypes among transitional and naïve cells (Table 4). No correlation was observed between the percentage of cytokine‐producing B‐cell subsets with either CXCL13 or NfL levels in either patient group (Supporting Information 7: Table S4).

**Table 4:** Correlation between CXCL13, NfL, and RRMS‐derived B‐cell subsets.

B cell subsets	CXCL13 (pg/mL)				NfL (pg/mL)			
	Stable		Unstable		Stable		Unstable	
	*R* ^a^	*p*‐Value	*R* ^a^	*p*‐Value	*R* ^a^	*p*‐Value	*R* ^a^	*p*‐Value
Total	0.1019	0.6711	0.2509	0.2231	0.2617	0.3452	0.2891	0.4191
HLA‐DR^+^ IgD^+^	0.1488	0.4419	0.06821	0.8119	0.3019	0.1050	0.1897	0.6711
HLA‐DR^+^ IgD^−^	0.2614	0.1906	0.5341	0.0521	0.3043	0.1020	0.3011	0.2311
HLA‐DR^−^ IgD^+^	−0.0591	0.7610	−0.4033	0.1966	−0.1786	0.3450	−0.2091	0.4891
Transitional	0.2711	0.3117	0.2713	0.3122	0.2891	0.1735	0.2034	0.3984
HLA‐DR^+^ IgD^+^	0.1971	0.3058	0.2617	0.3452	0.1945	0.3030	0.2152	0.4376
HLA‐DR^+^ IgD^−^	0.2724	0.1528	0.3900	0.1508	0.1098	0.5076	0.0207	0.0419
HLA‐DR^−^ IgD^+^	−0.071	0.7108	−0.4163	0.0746	**−0.4797**	**0.0073**	**−0.5155**	**0.0466**
Naïve	0.1076	0.6118	0.1878	0.4028	0.1993	0.2876	0.2616	0.4871
HLA‐DR^+^ IgD^+^	0.0967	0.6399	0.073	0.7951	0.1742	0.3572	0.1190	0.6644
HLA‐DR^+^ IgD^−^	0.0342	0.8600	0.3809	0.3081	0.097	0.6090	0.3565	0.1905
HLA‐DR^−^ IgD^+^	−0.1575	0.4145	−0.2256	0.9318	**−0.3861**	**0.0473**	**−0.5037**	**0.0376**
Plasmablast	**0.3716**	**0.0451**	**0.4874**	**0.0461**	0.1755	0.3781	0.1021	0.7833
CD138^+^	**0.4170**	**0.0366**	**0.5811**	**0.0221**	**0.3674**	**0.0481**	**0.5213**	**0.0402**
HLA‐DR^+^ IgD^+^	0.2030	0.2897	0.0289	0.9206	0.0346	0.8559	0.0045	0.9882
HLA‐DR^+^ IgD^−^	0.3417	0.0797	0.1957	0.4812	**0.3911**	**0.0322**	**0.7911**	**0.0017**
HLA‐DR^−^ IgD^+^	0.1061	0.9565	0.2786	0.3012	0.1954	0.3008	0.0193	0.9528
Memory								
CD27^−^CD38^−^	0.1705	0.3766	0.3012	0.2415	0.0817	0.6019	0.0712	0.7984
HLA‐DR^+^ IgD^+^	0.3096	0.1022	0.2306	0.4055	0.0346	0.8559	0.0045	0.9882
HLA‐DR^+^ IgD^−^	**0.3603**	**0.0473**	**0.4918**	**0.03871**	0.0131	0.9480	0.2735	0.3201
HLA‐DR^−^ IgD^+^	0.2383	0.2132	0.1981	0.5133	0.1954	0.3008	0.0193	0.9428
CD27^+^CD38^+^	0.1611	0.4510	0.2813	0.2177	0.2613	0.1891	0.2113	0.3981
HLA‐DR^+^ IgD^+^	0.0711	0.7067	0.3000	0.2767	0.2820	0.1310	0.1134	0.3782
HLA‐DR^+^ IgD^−^	**0.4087**	**0.0324**	**0.5429**	**0.0391**	0.3015	0.1054	0.1952	0.4753
HLA‐DR^−^ IgD^+^	0.1077	0.4577	0.1311	0.4330	0.1221	0.3008	0.2496	0.3660
CD27^+^CD38^−^	0.1705	0.3766	0.2014	0.3678	0.1456	0.4067	0.3093	0.2517
HLA‐DR^+^ IgD^+^	0.1205	0.5336	0.1040	0.7105	0.0562	0.7721	0.4606	0.0851
HLA‐DR^+^ IgD^−^	**0.3945**	**0.0367**	**0.4899**	**0.0413**	0.2126	0.2682	0.4878	0.0668
HLA‐DR^−^ IgD^+^	0.1349	0.4883	0.2498	0.3662	0.1359	0.4741	0.3110	0.2560

*Note:* In RRMS patients, the percentage of circulating total B cells (CD19^+^) and different B cell subsets [Transitional (CD38^++^ CD27^−^), naïve (CD38^+^ CD27^−^), total and CD138^+^ plasmablasts (CD38^++^CD27^++^) and memory cells (CD38^−^CD27^−^; CD38^+^ CD27^+^ and CD38^−^CD27^+^)], determined by flow cytometry, was correlated with the plasma levels of CXCL13 and NfL, assayed by ELISA. *ρ* = Spearman’s rank correlation coefficient. Significance *p* < 0.05. Statistically *p*‐values (*p* < 0.05) are highlighted in the bold.

^a^95% confidence intervals were estimated using the percentile bootstrap method with 2000 resamples.

## 4. Discussion

Although RRMS patients evolve through acute episodes of neurological impairment followed by total or partial remission, unfortunately most patients progress to the secondary neurodegenerative form (SPMS) despite DMT. This form is characterized by progressive neuroimage worsening and accumulation of irreversible physical and cognitive impairments due to neurodegeneration. The recent identification of B‐cell‐rich eFLs in the meninges underlying SPMS lesions suggests B‐cell involvement in the neurodegenerative process [11–14, 29]. Interestingly, our findings suggest that differential expression of HLA‐DR and IgD, as well as IL‐10 and IL‐17, on RRMS‐derived B‐cell subsets may indicate risk of disease progression.

It is known that most DMTs targeting effector/memory T‐cell lymphocytes [30] are considered the primary immune cells involved in demyelination in RRMS [31]. Nonetheless, therapies targeting B‐cell depletion produce significant clinical benefits, which have been associated with decreased pro‐inflammatory CD4^+^ and CD8^+^ T‐cell subtypes [32, 32]. Furthermore, some DMTs that modulate T‐cell functions also appear to target B‐cell subsets. Ramgolam et al. [33] demonstrated that treatment of RRMS patients with IFN‐β‐1β diminished the immunogenicity of B cells, identified by lower CD40 and CD80 expression and reduced production of GM‐CSF, IL‐1β and IL‐23 cytokines, while elevating IL‐10^+^ B‐cell frequency. Hâusler et al. [34] also found an increase in IL‐10^+^ B‐cell frequency, associated with decreased transitional cells and plasmablast frequency in RRMS patients treated with AG. Indeed, treatment of RRMS with DMF has been reported to reduce memory B cells [35, 36] and plasmablasts [34] but elevate transitional and IL‐10^+^ B‐cell frequency. RRMS patients undergoing FGL therapy showed elevated percentages of circulating plasmablasts, IL‐10^+^ B cells, and particularly transitional cells [37]. Finally, NATz has been shown to increase the percentage of different memory B‐cell subsets (CD27^+^CD38^+^, CD27^+^CD38^−^ and CD27^−^CD38^−^) but reduce plasmablasts and IgG titers in RRMS patients [38]. Also, NATz failure was associated with diminished circulating memory B‐cell subsets but elevated transitional and plasmablast cells [38]. Altogether, the studies above showed that these drugs preferentially target memory B cells, plasmablast, transitional, and IL‐10^+^ B cells. In our cohort, no consistent pattern across MS therapies for any of the B‐cell populations analyzed was found to be significant, probably due to our small sample size and/or the longer treatment time our patients underwent when compared with those recruited in the other studies.

Here, compared with healthy individuals, pwMS showed a higher proportion of total plasmablasts, with no difference in the other B‐cell subsets. Nonetheless, the analysis of the expression of other biological markers showed that an imbalance of different B‐cell subsets correlated with disease severity, regardless of DMT.

Higher frequency of HLA‐DR^+^, either IgD^+^ or IgD^−^, and most memory CD27^+^ and CD27^−^ B‐cell subsets were observed in pwMS by comparison with the control group. Regarding memory B cells, Negron et al. [8] found that the massive presence of eFLs in meningeal areas adjacent to neurodegenerative lesions in SPMS patients correlated with an abnormally expanded memory IgD^−^CD27^−^ B‐cell subset. Interestingly, a similar B‐cell phenotype, HLA‐DR^+^IgD^−^CD27^−^CD38^−^ was associated with higher risk for new relapses during the 1‐year follow‐up. For human memory B cells, loss of CD27 expression indicates differentiation and maturation [39, 40]. Therefore, circulating CD27^+^ B cells possibly become CD27^−^ cells when they are reactivated following a new relapse. Since studies have shown that the frequency of relapses elevates the risk of MS progression [41, 42], the correlation between HLA‐DR^+^IgD^−^CD27^+^CD38^+^ memory B‐cell frequency and severity of neurological impairment, determined by EDSS score, may be associated, at least in part, with the ability of CD27^+^IgD^−^ cells to become CD27^−^ ones. Interestingly, in the present study, plasma CXCL13 levels not only positively correlated with HLA‐DR^+^IgD^+^ memory B‐cell frequency but were also associated with occurrences of new relapses.

Beyond HLA‐DR^+^ memory B cells, disease activity positively correlated with total and CD138^+^plasmablasts. The frequency of plasmablast with HLA‐DR^+^IgD^−^ or CD138^+^ phenotypes was associated with both EDSS score and clinical activity. Plasmablasts, either CD138^+^ or HLA‐DR^+^IgD^−^ subsets, are a precursor to plasma cells that migrate to bone marrow and LFs from different secondary lymphoid organs [43, 44]. In lupus, CD138 expression and high HLA‐DR levels on plasmablasts correlate with disease activity and anti‐dsDNA antibody titers [45]. Moreover, in the present study, plasma CXCL13 levels positively correlated with CD138^+^ plasmablast.

Through CXCR5 expression, B cells can migrate to different tissues in response to chemokines, such as CXCL13 [46]. CXCL13, found to be elevated in the CSF and plasma of RRMS patients, correlated with the presence of B cells and plasmablasts [47]. Disano et al. [48] demonstrated that the chemokine CXCL13, by favoring eFL formation, can be an excellent biomarker of MS activity. Moreover, we observed a positive correlation between CD138^+^ and HLA‐DR^+^IgD^−^ plasmablasts and plasma NfL levels, a neuro‐specific marker of disease activity and neuroaxonal damage in MS [13].

In contrast to HLA‐DR^+^IgD^−^ cells, the presence of HLA‐DR^−^IgD^+^ cells was associated with less severe disease. Here, higher frequency of HLA‐DR^−^IgD^+^ memory B cells at baseline correlated with clinically stable MS. Although a lower proportion of HLA‐DR^−^IgD^+^ transitional cells was observed in pwMS than the control group, these cells were negatively associated with both neurological disabilities and occurrence of new relapses during follow‐up. Additionally, a higher percentage of HLA‐DR^−^IgD^+^ naïve cells was also observed among nonrelapsed patients. Also, plasma NfL levels negatively correlated with the presence of transitional and naïve B cells with HLA‐DR^−^IgD^+^ phenotype.

Given that HLA‐DR signaling amplifies B‐cell activation [49], we believe that the expansion of anergic HLA‐DR^−^ IgD^+^ B‐cell subsets is associated with better MS prognosis. Moreover, unlike IgA, IgE, IgG, and IgM, which are widely studied, the role of IgD is still not well understood [50, 51]. Some authors have found that high IgD levels on the B‐cell surface, associated with lower IgM expression, could be indicative of anergic lymphocytes in peripheral blood and secondary lymphoid tissues, most of them self‐reactive cells, leading to a dysfunctional response to self‐antigens [50, 51]. Although we did not evaluate IgM expression, more intense IgD expression according to B‐cell subset was observed in clinically stable RRMS patients.

The absence of HLA‐DR on MS‐related IgD^+^ B‐cell subsets, if confirmed in a larger patient cohort, may indicate that this immune regulation does not depend on their role as antigen‐presenting cells to CD4^+^ T cells but rather on their ability to produce anti‐inflammatory cytokines, such as IL‐10 [52].

Classically, cytokines like IL‐17 and IL‐10, mainly produced by effector (IL‐17) and regulatory (IL‐10) lymphocytes, have been associated with MS disease activity and control, respectively [53]. In the present study, although the effect of DMTs in inhibiting pro‐inflammatory cytokine production [32–38] may explain the lower frequency of IL‐17^+^ B‐cell subsets in pwMS as compared with healthy individuals, the frequency of IL‐17^+^IL‐10^−^ B‐cell subsets was associated with clinical relapses. Also, the presence of IL‐17^+^IL‐10^−^ plasmablasts and IL‐17^+^ memory CD27^+^CD28^+^ B cells correlated positively with EDSS score. On the other hand, the presence of an elevated percentage of IL‐17^−^IL‐10^+^ transitional B cells was associated with both less neurological damage (a lower EDSS score) and absence of new relapses.

In humans, transitional cells are closely related to IL‐10‐producing regulatory B cells (Bregs) in terms of phenotypical and functional similarities [2]. As reported by Zhou et al. [54], the main functions of transitional B cells are able to suppress both proliferation of autoreactive CD4^+^ T cells and inhibit Th1 and Th17 cell differentiation, thus limiting excessive production of pro‐inflammatory cytokines, such as IL‐17. Moreover, transitional cells promote the conversion of effector CD4^+^ T cells into regulatory T cells (Tregs). Many of these regulatory immune functions have been associated with IL‐10 production by transitional cells that are able to inhibit CD8^+^ T‐cell responses [55]. However, damage to transitional cells has been observed in autoimmune diseases, including MS [56, 57], neuromyelitis optica spectrum disorders (NMOSDs) [58], and lupus [59]. In MS, fewer transitional cells were detected in patients with clinically isolated syndrome (CIS) than in healthy individuals, and the frequency of transitional cells was increased after related treatments, such as FGL [60–62]. Transitional cells in the peripheral blood of pwMS undergoing treatment with FGL produced more IL‐10 and less TNF‐α than memory and mature naïve B cells and expressed low CD80 levels [60]. Clinical control of relapses by therapy with IFN‐β, DMF, and FGL correlates with increased frequency of circulating IL‐10^+^ transitional and IL‐10^+^ naïve B cells and reduced proportion of memory IgD^−^ B cells [63–66]. Moreover, a study by Grutzke et al. [62] found that IL‐10‐producing B cells, induced by FGL treatment, were able to migrate into the CNS and reduce disease severity. Currently, our group is dedicated to analyzing the frequency of different RRMS‐derived B‐cell subtypes just before and 1 year after initiating DMT.

In addition to transitional cells, IL‐17^−^IL‐10^+^ plasmablasts were inversely correlated with neurological impairment in the present study. In an experimental MS model, named Experimental Autoimmune Encephalomyelitis (EAE), expansion of IL‐10^+^ plasmablasts attenuated disease severity, which was associated with decreased infiltration of pathogenic T cells into the CNS of the animals [67]. Therefore, the ability of DMT to expand tolerogenic B‐cell phenotypes, such as HLA‐DR^−^IgD^+^ and IL‐17^−^IL‐10^+^, mainly among transitional and plasmablast cells, may contribute to achieving a more stable clinical evolution of the disease, known as “no evidence of disease activity (*NEDA*)” [68].

Although these findings are interesting, in the present study, the B cells were stimulated by a nonphysiological, receptor‐independent B‐cell activator, which does not allow to determine the fraction of dysregulated B cells directed against myelin antigens. Additionally, independent external validation in larger, multicenter cohorts will be required to confirm the reproducibility and generalizability of these findings.

In addition, it is possible that the difference in B‐cell subsets between stable and unstable MS patients is related to the ethnicity. As demonstrated in Table 1, the majority of relapsed patients were Afrodescendent. As compared with Caucasian individuals, MS is more severe and less responsive to DMT treatment in Afrodescendent, including among Brazilian patients [69–72].

By using similar surface markers (CD27, CD38, IgD, and CD138), Telesford et al. [73] demonstrated significant difference in circulating B‐cell subsets in natalizumab‐treated MS patient according to self‐reported ethnicity. As compared with Caucasians, Afrodescent showed an increased baseline frequency of memory B cells, CD138^+^plasmablasts, and elevated systemic and CSF antineuron IgG [73]. Unfortunately, those authors did not evaluate the percentage of IgD^+^HLA‐DR^−^ B cells. Therefore, it is probably that elevated prevalence of Afrodescent in unstable patients contribute to perfect, or near‐perfect, predictive performance between B‐cell subsets and clinical activity of MS observed in our cohort.

## 5. Conclusions

Despite our small sample size, the data presented here are original and indicate, for the first time, that accumulation of B‐cell subsets differently expressing HLA‐DR, IgD, IL‐17, and IL‐10, associated with the expression of CD138 on plasmablasts, could be effective biomarkers for differentiating DMT‐treated RRMS patients at low‐ or high‐risk of therapy failure and disease evolution. Notably, MS severity was associated with the presence of CD138^+^ plasmablast and IL‐17^+^IL‐10^−^, HLA‐DR^+^IgD^−^ cell phenotypes among plasmablast, transitional, and memory CD27^+^CD38^+^B‐cell subsets. On the other hand, stable DMT‐treated patients showed higher frequency of circulating transitional and plasmablasts with HLA‐DR^−^IgD^+^ and IL‐17^−^IL‐10^+^ phenotypes.

## Funding

This work was supported by Fundação de Amparo à Pesquisa Carlos Chagas Filho (FAPERJ (Grants E‐26/202.940/2017 and E‐26/202.726/2019) and Conselho Nacional de Desenvolvimento Científico e Tecnológico (CNPq, Grant 301.780/2017‐0).

## Disclosure

The authors declare that no generative AI was used in the creation of this manuscript.

## Ethics Statement

The study was approved by the Ethics Committee for Research on Human Subjects of the Federal University of the State of Rio de Janeiro (UNIRIO) (CAAE number: 43009015.6.0000.5258). All samples were collected only after written informed consent was obtained from each individual.

## Conflicts of Interest

The authors declare no conflicts of interest.

## Supporting Information

Additional supporting information can be found online in the Supporting Information section.

## Supporting information


**Supporting Information 1** Figure S1: Flow cytometry supporting information and representative dot‐plots of different B cell (CD19^+^) subsets in healthy and RRMS patients.

**Supporting Information 2** Figure S2: Analysis of the different RRMS‐derived B cell subsets according to treatment. In DMT‐treated RRMS patients.

**Supporting Information 3** Figure S3: The stimulation‐induced IL‐17 and IL‐10 response by B cells in healthy and RRMS patients.

**Supporting Information 4** Table S1: Multivariate firth logistic regression.

**Supporting Information 5** Table S2: ROC analysis of firth logistic regression models.

**Supporting Information 6** Table S3: LOOCV validation of firth logistic regression models.

**Supporting Information 7** Table S4: Correlation between CXCL13, NfL and cytokine‐producing B cell subsets from MS patients.

**Supporting Information 8** Figure S4: The frequency of total CXCR5^+^ B cells in healthy and stable and relapsed RRMS patients.

## Data Availability

The datasets generated during and/or analyzed in the current study are available from the corresponding author upon reasonable request.
