# Supplementary material for: Imbalance of Circulating B‐Cell Subsets Was Associated With Higher Risk of Relapses in RRMS Patients
Source: J Immunol Res. 2026 Sep 22;2026:7341319. doi: 10.1155/jimr/7341319 (PMC13598429; doi:10.1155/jimr/7341319)
Supplement: Supplementary file 1 [file JIMR-2026-7341319-s004.docx]

**Table S2. ROC Analysis of Firth Logistic Regression Models**

| **B cells** | **AUC** | **AUC_IC95** | **Cutoff** | **Sensitivity** | **Specificity** | **Accuracy** |
| --- | --- | --- | --- | --- | --- | --- |
| *HLA-DR-IgD^+^ cell subset* |  |  |  |  |  |  |
| Transitional | 0.986 | 0.961 - 1 | 0.321 | 1.000 | 0.893 | 0.930 |
| Naive | 1.000 | 1 - 1 | 0.543 | 1.000 | 1.000 | 1.000 |
| Plasmabast | 1.000 | 1 - 1 | 0.581 | 1.000 | 1.000 | 1.000 |
| CD27^-^CD38^-^ memory | 0.993 | 0.979 - 1 | 0.360 | 1.000 | 0.931 | 0.955 |
| CD27^+^CD38^-^ memory | 1.000 | 1 - 1 | 0.665 | 1.000 | 1.000 | 1.000 |
| CD27^+^CD38+ memory | 1.000 | 1 - 1 | 0.479 | 1.000 | 1.000 | 1.000 |
| *IL-17^+^IL-10- cell subset* |  |  |  |  |  |  |
| Transitional | 0.853 | 0.745 - 0.962 | 0.194 | 1.000 | 0.600 | 0.733 |
| Naive | 0.913 | 0.832 - 0.995 | 0.274 | 0.867 | 0.833 | 0.844 |
| Plasmabast | 1.000 | 1 - 1 | 0.545 | 1.000 | 1.000 | 1.000 |
| CD27^-^CD38^-^ memory | 0.973 | 0.937 - 1 | 0.237 | 1.000 | 0.867 | 0.911 |
| CD27^+^CD38^+^ memory | 0.956 | 0.903 - 1 | 0.187 | 1.000 | 0.800 | 0.867 |
| CD27^+^CD38^-^ memory | 0.996 | 0.985 - 1 | 0.517 | 0.933 | 1.000 | 0.978 |
| *IL-17-IL-10^+^ cell subset* |  |  |  |  |  |  |
| Transitional | 1.000 | 1 - 1 | 0.641 | 1.000 | 1.000 | 1.000 |
| Plasmabast | 0.929 | 0.846 - 1 | 0.592 | 0.800 | 0.967 | 0.911 |
| *MFI of HLA-DR per cell subset* |  |  |  |  |  |  |
| Transitional | 1.000 | 1 - 1 | 0.326 | 1.000 | 1.000 | 1.000 |
| Plasmabast | 0.974 | 0.938 - 1 | 0.219 | 1.000 | 0.857 | 0.907 |
| CD27^+^CD38^-^ memory | 0.944 | 0.865 - 1 | 0.293 | 0.867 | 0.967 | 0.933 |
| CD27^-^CD38^-^ memory | 0.984 | 0.958 - 1 | 0.458 | 0.867 | 1.000 | 0.956 |
| *MFI of IgD per cell subset* |  |  |  |  |  |  |
| Transitional | 0.864 | 0.76 - 0.969 | 0.277 | 1.000 | 0.667 | 0.778 |
| Naive | 0.904 | 0.81 - 0.997 | 0.329 | 0.933 | 0.778 | 0.833 |
| Plasmabast | 0.949 | 0.893 - 1 | 0.335 | 1.000 | 0.800 | 0.867 |
| CD27^+^CD38^+^ memory | 0.940 | 0.87 - 1 | 0.331 | 0.933 | 0.900 | 0.911 |
| *Plasmabast MFI CD138* | 0.983 | 0.955 - 1 | 0.322 | 0.933 | 0.964 | 0.953 |
| *Plasma CXCL13* | 0.807 | 0.674 - 0.94 | 0.293 | 0.867 | 0.690 | 0.750 |

ROC: Receiver Operating Characteristic; AUC: Area under the curve
