# Supplementary material for: Imbalance of Circulating B‐Cell Subsets Was Associated With Higher Risk of Relapses in RRMS Patients
Source: J Immunol Res. 2026 Sep 22;2026:7341319. doi: 10.1155/jimr/7341319 (PMC13598429; doi:10.1155/jimr/7341319)
Supplement: Supplementary file 2 [file JIMR-2026-7341319-s005.docx]

**Table S3. LOOCV Validation of Firth Logistic Regression Models.**

| **B cells** | **LOOCV_AUC** | **LOOCV_AUC_IC95** | **Cutoff** | **Sensitivity** | **Specificity** | **Accuracy** |
| --- | --- | --- | --- | --- | --- | --- |
| *HLA-DR-IgD^+^ cell subset* |  |  |  |  |  |  |
| Transitional | 0.933 | 0.863 - 1 | 0.089 | 1.000 | 0.714 | 0.814 |
| Naive | 0.986 | 0.956 - 1 | 0.599 | 1.000 | 0.964 | 0.977 |
| Plasmabast | 0.998 | 0.991 - 1 | 0.523 | 1.000 | 0.966 | 0.977 |
| CD27^-^CD38^-^ memory | 0.947 | 0.871 - 1 | 0.282 | 1.000 | 0.931 | 0.955 |
| CD27^+^CD38^+^ memory | 1.000 | 1 - 1 | 0.421 | 1.000 | 1.000 | 1.000 |
| CD27^+^CD38^-^ memory | 0.975 | 0.936 - 1 | 0.491 | 1.000 | 0.931 | 0.955 |
| *IL-17^+^IL-10- cell subset* |  |  |  |  |  |  |
| Transitional | 0.749 | 0.596 - 0.902 | 0.263 | 0.800 | 0.633 | 0.689 |
| Naive I | 0.809 | 0.664 - 0.954 | 0.245 | 0.800 | 0.700 | 0.733 |
| Plasmabast | 0.995 | 0.984 - 1 | 0.503 | 1.000 | 0.967 | 0.977 |
| CD27^-^CD38^-^ memory | 0.947 | 0.888 - 1 | 0.155 | 1.000 | 0.767 | 0.844 |
| CD27^+^CD38^+^ memory | 0.909 | 0.819 - 0.998 | 0.410 | 0.800 | 0.900 | 0.867 |
| CD27^+^CD38^-^ memory | 0.982 | 0.946 - 1 | 0.580 | 0.933 | 1.000 | 0.978 |
| *IL-17-IL-10^+^ cell subset* |  |  |  |  |  |  |
| Transitional | 0.980 | 0.95 - 1 | 0.284 | 1.000 | 0.900 | 0.933 |
| Plasmabast | 0.853 | 0.727 - 0.979 | 0.366 | 0.867 | 0.767 | 0.800 |
| *MFI of HLA-DR per cell subset* |  |  |  |  |  |  |
| Transitional | 0.778 | 0.644 - 0.911 | 0.179 | 1.000 | 0.533 | 0.689 |
| Plasmabast | 0.902 | 0.793 - 1 | 0.221 | 0.933 | 0.857 | 0.884 |
| CD27^-^CD38^-^ memory | 0.920 | 0.816 - 1 | 0.399 | 0.867 | 0.933 | 0.911 |
| CD27^+^CD38- memory | 0.856 | 0.716 - 0.996 | 0.200 | 0.867 | 0.767 | 0.800 |
| *MFI of IgD per cell subset* |  |  |  |  |  |  |
| Transitional | 0.978 | 0.94 - 1 | 0.464 | 0.933 | 0.967 | 0.956 |
| Naive | 0.802 | 0.657 - 0.948 | 0.293 | 0.933 | 0.630 | 0.738 |
| Plasmabast | 0.957 | 0.9 - 1 | 0.310 | 0.867 | 0.964 | 0.930 |
| CD27^+^CD38^+^ memory | 0.907 | 0.819 - 0.994 | 0.203 | 0.933 | 0.767 | 0.822 |
| *MFI of CD138 per plasmablast* | 0.957 | 0.9 - 1 | 0.310 | 0.967 | 0.964 | 0.930 |
| *CXCL13* | 0.655 | 0.472 - 0.838 | 0.267 | 0.800 | 0.517 | 0.614 |

LOOCV: leave-one-out cross-validation
