# Supplementary material for: Imbalance of Circulating B‐Cell Subsets Was Associated With Higher Risk of Relapses in RRMS Patients
Source: J Immunol Res. 2026 Sep 22;2026:7341319. doi: 10.1155/jimr/7341319 (PMC13598429; doi:10.1155/jimr/7341319)
Supplement: Supplementary file 3 [file JIMR-2026-7341319-s006.docx]

**Table S4. Correlation between CXCL13, NfL and cytokine-producing B cell subsets from MS patients.**

|  | CXCL13 (pg/mL) | | | | NfL (pg/mL) | | | |
| --- | --- | --- | --- | --- | --- | --- | --- | --- |
|  | Stable | | Unstable | | Stable | | Unstable | |
|  | R^1^ | P value | R^1^ | P value | R^1^ | P value | R^1^ | P value |
| Total |  |  |  |  |  |  |  |  |
| IL-17^+^ IL-10^-^ | 0.2123 | 0.3610 | 0.4448 | 0.0975 | 0.2661 | 0.1630 | 0.3071 | 0.2650 |
| IL-17^-^ IL-10^+^ | 0.2391 | 0.2031 | 0.3105 | 0.2481 | 0.1350 | 0.4849 | 0.2151 | 0.4400 |
| IL-17^+^ IL-10^+^ | 0. 1776 | 0.3477 | 0.2746 | 0.1419 | 0.1088 | 0.5451 | 0.0775 | 0.7270 |
|  |  |  |  |  |  |  |  |  |
| Transitional |  |  |  |  |  |  |  |  |
| IL-17^+^ IL-10^-^ | 0. 2899 | 0.1201 | 0.5154 | 0.0613 | 0.0283 | 0.8839 | 0.4429 | 0.1002 |
| IL-17^-^ IL-10^+^ | -0.3001 | 0.1013 | 0.1896 | 0.4928 | 0.1047 | 0.5887 | 0.071 | 0.8025 |
| IL-17^+^ IL-10^+^ | -0.2346 | 0.2122 | 0.4169 | 0.1220 | -0.3488 | 0.0637 | 0.4482 | 0.0952 |
|  |  |  |  |  |  |  |  |  |
| Naïve |  |  |  |  |  |  |  |  |
| IL-17^+^ IL-10^-^ | 0. 2075 | 0.2711 | 0.1694 | 0.2711 | 0.0813 | 0.9666 | 0.4286 | 0.1177 |
| IL-17^-^ IL-10^+^ | 0.2189 | 0.2453 | 0.1479 | 0.2453 | 0.2348 | 0.2202 | 0.2440 | 0.3998 |
| IL-17^+^ IL-10^+^ | 0.1339 | 0.4837 | 0.5119 | 0.4837 | 0.1563 | 0.4164 | 0.3295 | 0.2286 |
|  |  |  |  |  |  |  |  |  |
| Plasmablast |  |  |  |  |  |  |  |  |
| IL-17^+^ IL-10^-^ | 0.2914 | 0.1183 | 0.4202 | 0.1192 | 0.029 | 0.8778 | 0.4770 | 0.0713 |
| IL-17^-^ IL-10^+^ | 0.3464 | 0.0608 | 0.077 | 0.8071 | 0.0341 | 0.9858 | -0.1362 | 0.6282 |
| IL-17^+^ IL-10^+^ | 0.2118 | 0.2612 | 0.4820 | 0.0706 | 0.0137 | 0.9433 | -0.2656 | 0.3350 |
|  |  |  |  |  |  |  |  |  |
| Memory |  |  |  |  |  |  |  |  |
| CD27^-^CD38^-^ |  |  |  |  |  |  |  |  |
| IL-17^+^ IL-10^-^ | 0.3285 | 0.0991 | 0.4018 | 0.1378 | 0.3435 | 0.0681 | 0.0840 | 0.7654 |
| IL-17^-^ IL-10^+^ | 0.1318 | 0.4876 | 0.3568 | 0.1907 | -0.3613 | 0.0540 | 0.017 | 0.9745 |
| IL-17^+^ IL-10^+^ | 0.1277 | 0.5114 | 0.3246 | 0.2353 | -0.3725 | 0.0466 | 0.1037 | 0.7116 |
|  |  |  |  |  |  |  |  |  |
| CD27^+^CD38^+^ |  |  |  |  |  |  |  |  |
| IL-17^+^ IL-10^-^ | 0.2604 | 0.1647 | 0.2663 | 0.3339 | 0.093 | 0.6315 | 0.3500 | 0.2012 |
| IL-17^-^ IL-10^+^ | 0.2260 | 0.2298 | 0.3276 | 0.2309 | -0.2176 | -0.2567 | 0.2116 | 0.4461 |
| IL-17^+^ IL-10^+^ | 0.3308 | 0.0742 | 0.1509 | 0.5864 | -0.3368 | -0.0740 | 0.036 | 0.8995 |
|  |  |  |  |  |  |  |  |  |
| CD27^+^CD38^-^ |  |  |  |  |  |  |  |  |
| IL-17^+^ IL-10^-^ | 0.3301 | 0.0714 | 0.2831 | 0.3194 | 0.1208 | 0,5325 | 0.4043 | 0.1354 |
| IL-17^-^ IL-10^+^ | 0.099 | 0.6032 | 0.088 | 0.7790 | 0.082 | 0.6740 | -0.5109 | 0.065 |
| IL-17^+^ IL-10^+^ | 0.092 | 0.6308 | 0.4532 | 0.0918 | 0.2128 | 0.2677 | -0.2521 | 0.3681 |

In stable and unstable RRMS patients, the percentage of IL-17 and IL-10 producing B cells among toral (CD19^+^), Transitional (CD38^++^ CD27^-^), naïve (CD38^+^ CD27^-^), plasmablasts (CD38^++^CD27^+^+) and memory cell subsets (CD38^-^CD27^-^; CD38^+^ CD27^+^ and CD38^-^CD27^+^), determined by flow cytometry, was correlated with the plasma levels of CXCL13 and NfL, assayed by ELISA. *^1^*95% confidence intervals were estimated using the percentile bootstrap method with 2,000 resamples. *ρ= Spearman's rank correlation coefficient.* Significancy P<0.05.
