# Supplementary material for: Imbalance of Circulating B‐Cell Subsets Was Associated With Higher Risk of Relapses in RRMS Patients
Source: J Immunol Res. 2026 Sep 22;2026:7341319. doi: 10.1155/jimr/7341319 (PMC13598429; doi:10.1155/jimr/7341319)
Supplement: Supplementary file 4 [file JIMR-2026-7341319-s008.docx]

### Table S1. Multivariate Firth Logistic Regression

| **B cells** | **Adjusted OR (CI 95%)** | p-value |
| --- | --- | --- |
| *HLA-DR^+^IgD- cell subset* |  |  |
| Transitional | 1.36 (1.12–1.65) | 0.002 |
| Naïve | 1.10 (1.03–1.18) | 0.003 |
| Plasmablast | 1.42 (1.04–1.93) | 0.026 |
| CD27-CD38- memory | 1.24 (1.06–1.46) | 0.007 |
| CD27^+^CD38^+^ memory | 1.13 (1.05–1.22) | 0.002 |
| *CD138^+^ Plasmabast* | 1.11 (1.02–1.21) | 0.015 |
| *HLA-DR-IgD^+^ cell subset* |  |  |
| Transitional | 0.64 (0.45–0.91) | 0.014 |
| Naive | 0.60 (0.44–0.81) | <0.001 |
| Plasmabast | 0.68 (0.52–0.88) | 0.004 |
| CD27-CD38- memory | 0.53 (0.30–0.92) | 0.025 |
| CD27^+^CD38^+^ memory | 0.68 (0.54–0.86) | 0.001 |
| CD27^+^CD38- memory | 0.69 (0.55–0.88) | 0.002 |
| *IL-17^+^IL-10- cell subset* |  |  |
| Transitional | 1.49 (1.14–1.94) | 0.004 |
| Plasmabast | 2.54 (1.35–4.78) | 0.004 |
| CD27-CD38- memory | 2.52 (1.39–4.59) | 0.002 |
| CD27^+^CD38^+^ memory | 1.68 (1.24–2.28) | <0.001 |
| CD27^+^CD38- memory | 2.07 (1.29–3.34) | 0.003 |
| *IL-17-IL-10^+^ cell subset* |  |  |
| Transitional | 0.37 (0.19–0.71) | 0.003 |
| Plasmabast | 0.46 (0.28–0.75) | 0.002 |
| CD27^+^CD38^+^ memory | 0.84 (0.67–1.05) | 0.119 |
| *MFI of HLA-DR per cell subset* |  |  |
| Transitional | 1.00 (1.00–1.00) | 0.011 |
| Plasmabast | 1.00 (1.00–1.00) | 0.001 |
| CD27-CD38- memory | 1.00 (1.00–1.00) | 0.002 |
| CD27^+^CD38- memory | 1.00 (1.00–1.00) | 0.011 |
| *MFI of IgD per cell subset* |  |  |
| Naive | 1.00 (1.00–1.00) | 0.032 |
| Plasmabast | 1.00 (1.00–1.00) | 0.018 |
| CD27^+^CD38^+^ memory | 1.00 (1.00–1.00) | <0.001 |
|  |  |  |
| *MFI of CD138 per Plasmabast* | 1.00 (1.00–1.00) | 0.004 |
| *Plasma CXCL13* | 1.00 (1.00–1.01) | 0.030 |

Models adjusted for the same control variables: Age, EDSS, and Gender.
